# Database of Vascular Plants of Canada (VASCAN): a community contributed taxonomic checklist of all vascular plants of Canada, Saint Pierre and Miquelon, and Greenland

**DOI:** 10.3897/phytokeys.25.3100

**Published:** 2013-07-24

**Authors:** Peter Desmet, Luc Brouillet

**Affiliations:** 1 Université de Montréal Biodiversity Centre, 4101 rue Sherbrooke est, H1X2B2, Montreal, Canada

**Keywords:** VASCAN, Canadensys, Canada, Greenland, Saint Pierre and Miquelon, checklist, taxonomy, synonymy, hybrids, vernacular names, English, French, distribution, provinces, habit, open data

## Abstract

The Database of Vascular Plants of Canada or VASCAN (http://data.canadensys.net/vascan) is a comprehensive and curated checklist of all vascular plants reported in Canada, Greenland (Denmark), and Saint Pierre and Miquelon (France). VASCAN was developed at the Université de Montréal Biodiversity Centre and is maintained by a group of editors and contributors. For every core taxon in the checklist (species, subspecies, or variety), VASCAN provides the accepted scientific name, the accepted French and English vernacular names, and their synonyms/alternatives in Canada, as well as the distribution status (native, introduced, ephemeral, excluded, extirpated, doubtful or absent) of the plant for each province or territory, and the habit (tree, shrub, herb and/or vine) of the plant in Canada. For reported hybrids (nothotaxa or hybrid formulas) VASCAN also provides the hybrid parents, except if the parents of the hybrid do not occur in Canada. All taxa are linked to a classification. VASCAN refers to a source for all name, classification and distribution information.

All data have been released to the public domain under a CC0 waiver and are available through Canadensys and the Global Biodiversity Information Facility (GBIF). VASCAN is a service to the scientific community and the general public, including administrations, companies, and non-governmental organizations.

## Data published through

The Canadensys repository: http://dx.doi.org/10.5886/1bft7W5f

## Project details

### Project title

Database of Vascular Plants of Canada (VASCAN)

### Personnel

**Data compilation editors:** Luc Brouillet (Université de Montréal Biodiversity Centre): coordination, taxonomic and geographic compilation, Frédéric Coursol (Montreal Botanical Garden): taxonomic and geographic compilation, Susan Meades (Northern Ontario Plant Database): taxonomic compilation, Marc Favreau (Translation Bureau, Public Works and Government Services Canada): French vernacular names compilation, Marilyn Anions (botanist, Ottawa): English vernacular names compilation.

**Development:** Peter Desmet (Université de Montréal Biodiversity Centre): coordination and web design, Pierre Bélisle (Université de Montréal Biodiversity Centre): development, Christian Gendreau (Université de Montréal Biodiversity Centre): development and maintenance, David Shorthouse (Université de Montréal Biodiversity Centre): coordination, Patrick O’Reilley (Université de Montréal): initial data import.

### Funding

Partial funding came from Parks Canada, the Canadian Biodiversity Information Facility (CBIF), NatureServe Canada, the Canadian Foundation for Innovation (CFI), and the Gouvernement du Québec (grant to the Université de Montréal Biodiversity Centre and Canadensys). Most of the compilation work, however, was contributed in kind by the home institution of each collaborator.

### Study area description

The study area occupies the northern half of North America (excluding Alaska). The area of Canada is 9,984,670 km^2^, of Greenland (or Kulaalit Nunaat, an autonomous country within the kingdom of Denmark) 2,166,086 km^2^, and Saint Pierre and Miquelon (collectivité territoriale, France) 242 km^2^. The latter is 20 km off the coast of Newfoundland’s Burin Peninsula and its characteristics are those of boreal eastern Canada. From west to east, the main physiographic regions are the Western Cordillera, the sedimentary Interior Plains, the Canadian and Greenland Shields (mostly igneous rocks), the sedimentary Great Lakes and St. Lawrences Lowlands, and the Appalachian Mountains. The sedimentary Hudson Bay Lowlands basin lies at the centre of the shield, a northern area of sedimentary plains and mountains. The Canadian Arctic borders the Arctic Ocean in northern Canada and northern Greenland. An ice cap covers 81% of Greenland.

The dominant vegetation type of the area is the boreal forest, which occupies much of Canada from Yukon and northeastern British Columbia to Newfoundland. To the north, Arctic tundra prevails: it can be divided into low Arctic (with a nearly continuous plant cover, sometimes shrubby) and high Arctic (including polar deserts); these types are the only ones found in Greenland. To the south of the boreal forest, from west to east, are the humid Pacific Coastal forest, the Cordilleran forest, the Prairie grasslands, the eastern temperate forests (southern Ontario and Quebec), and the Atlantic or Acadian forests.

The population of Canada is concentrated in a narrow belt along its border with the United States, where most of the impacts on ecosystems (urbanization, agriculture) is concentrated. Logging, mining, and hydroelectric development occur in the boreal forest, and mining is now rapidly developing in the Arctic. About 9.9% (Environment Canada 2011) of the terrestrial area of Canada is protected (7.5% according to the World Bank 2013) and 40% of Greenland. Based on the data in VASCAN, the area harbors a total of 5,124 vascular plant species, 3,829 native and 1,295 introduced (25% of the flora). Of the native species of Canada, 156 are considered legally at risk, with a further 34 of conservation concern (COSEWIC 2009+).

### Design description

The goal of the Database of Vascular Plants of Canada (VASCAN) is to provide an up-to-date, documented checklist of the names of vascular plants in Canada, Greenland, and Saint Pierre and Miquelon, both scientific and vernacular, and the distribution of the plants at the provincial/territorial level.

VASCAN was developed from the need to validate vascular plant name and distribution data from eastern Canada (Ontario and eastward), Greenland, and Saint Pierre and Miquelon for the Flora of North America project (FNA) and from the need to provide French vernacular names for taxa present in Quebec in the FNA. It expanded when Parks Canada wanted to harmonize the names from vascular plant species lists of its parks across the country. At the time we also realized that - aside from The Flora of Canada by Scoggan (1978-1979) that was in need of updating - not only was there no standardized scientific name list for the country - despite worthwhile efforts from Kartesz (1999) and USDA NRCS (2011) - but also no standardized source of Canadian English and French vernacular names. Names used for plants in English Canada are not necessarily those used in the United States, and thus U.S. sources were not always appropriate for this goal. Finally, several national organizations, such as Parks Canada, Forest Canada, the Committee on the Status of Endangered Wildlife in Canada (COSEWIC), and NatureServe Canada, expressed the need for a web-based list of Canadian taxa, with data on provincial/territorial distribution.

## Taxonomic coverage

**Figure 1.**
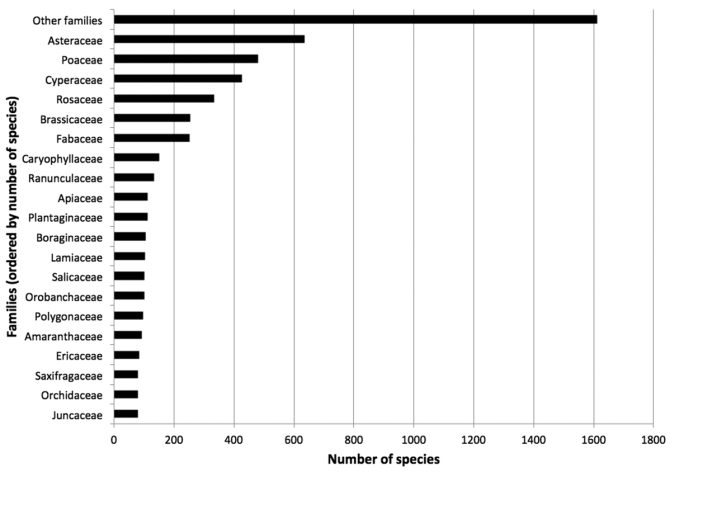
Taxonomic distribution of accepted species per family from the Database of Vascular Plants of Canada (VASCAN). The families are ordered by total number of species. Families with less than 80 species are grouped in ‘Other families’.

This checklist covers all vascular plants (*Equisetopsida*, *Tracheophyta*) reported in the area described in the section ‘Spatial Coverage’ (Figure 1). The core taxa considered are species, subspecies or varieties, and their hybrids. For these taxa, we provide synonyms, the accepted and alternative French and English vernacular names, and the habit (tree, shrub, herb and/or vine) of the plant in Canada. For reported hybrids (nothotaxa or hybrid formulas) we also indicate the hybrid parents, except if the parents of the hybrid do not occur in Canada. This core information is not provided for higher taxa, although the calculated distribution based on lower taxa can be consulted and downloaded from the VASCAN website (http://data.canadensys.net/vascan).

All taxa are linked to a classification: Chase and Reveal (2009) for the higher classification, Christenhusz et al. (2011a) for lycophytes, Smith et al. (2006) for monilophytes (modified in Rothfells et al. 2012), Christenhusz et al. (2011b) for the gymnosperms, and the Angiosperm Phylogeny Group (2009) for flowering plants. At the generic level and below, the Flora of North America Editorial Committee (1993+) is the main source of classification, unless taxonomic literature more recent than the volume published for a given taxon provides a taxonomy more reflective of current data. The source used is indicated for each taxon in the dataset.

The classification includes 16 ranks. They are, in hierarchical order: class, subclass, superorder, order, family, subfamily, tribe, subtribe, genus, subgenus, section, subsection, series, species, subspecies and variety. Varieties within subspecies are accepted, so quadrinomial names are present, but forms are not included.

### Taxonomic ranks

**Kingdom:** *Plantae*

**Class:** *Equisetopsida*

**Family:** *Acanthaceae*, *Acoraceae*, *Adoxaceae*, *Alismataceae*, *Amaranthaceae*, *Amaryllidaceae*, *Anacardiaceae*, *Annonaceae*, *Apiaceae*, *Apocynaceae*, *Aquifoliaceae*, *Araceae*, *Araliaceae*, *Aristolochiaceae*, *Asparagaceae*, *Aspleniaceae*, *Asteraceae*, *Athyriaceae*, *Balsaminaceae*, *Berberidaceae*, *Betulaceae*, *Bignoniaceae*, *Blechnaceae*, *Boraginaceae*, *Brassicaceae*, *Butomaceae*, *Buxaceae*, *Cabombaceae*, *Cactaceae*, *Campanulaceae*, *Cannabaceae*, *Caprifoliaceae*, *Caryophyllaceae*, *Celastraceae*, *Ceratophyllaceae*, *Cistaceae*, *Cleomaceae*, *Clethraceae*, *Colchicaceae*, *Commelinaceae*, *Convolvulaceae*, *Cornaceae*, *Crassulaceae*, *Cucurbitaceae*, *Cupressaceae*, *Cyperaceae*, *Cystopteridaceae*, *Dennstaedtiaceae*, *Diapensiaceae*, *Dioscoreaceae*, *Droseraceae*, *Dryopteridaceae*, *Elaeagnaceae*, *Elatinaceae*, *Equisetaceae*, *Ericaceae*, *Eriocaulaceae*, *Euphorbiaceae*, *Fabaceae*, *Fagaceae*, *Frankeniaceae*, *Gentianaceae*, *Geraniaceae*, *Grossulariaceae*, *Haemodoraceae*, *Haloragaceae*, *Hamamelidaceae*, *Hydrangeaceae*, *Hydrocharitaceae*, *Hymenophyllaceae*, *Hypericaceae*, *Hypoxidaceae*, *Iridaceae*, *Isoëtaceae*, *Juglandaceae*, *Juncaceae*, *Juncaginaceae*, *Lamiaceae*, *Lauraceae*, *Lentibulariaceae*, *Liliaceae*, *Limnanthaceae*, *Linaceae*, *Linderniaceae*, *Loasaceae*, *Loranthaceae*, *Lycopodiaceae*, *Lythraceae*, *Magnoliaceae*, *Malvaceae*, *Marsileaceae*, *Martyniaceae*, *Melanthiaceae*, *Melastomataceae*, *Menispermaceae*, *Menyanthaceae*, *Molluginaceae*, *Montiaceae*, *Moraceae*, *Myricaceae*, *Nartheciaceae*, *Nelumbonaceae*, *Nyctaginaceae*, *Nymphaeaceae*, *Oleaceae*, *Onagraceae*, *Onocleaceae*, *Ophioglossaceae*, *Orchidaceae*, *Orobanchaceae*, *Osmundaceae*, *Oxalidaceae*, *Paeoniaceae*, *Papaveraceae*, *Penthoraceae*, *Phrymaceae*, *Phytolaccaceae*, *Pinaceae*, *Plantaginaceae*, *Platanaceae*, *Plumbaginaceae*, *Poaceae*, *Podostemaceae*, *Polemoniaceae*, *Polygalaceae*, *Polygonaceae*, *Polypodiaceae*, *Pontederiaceae*, *Portulacaceae*, *Potamogetonaceae*, *Primulaceae*, *Pteridaceae*, *Ranunculaceae*, *Resedaceae*, *Rhamnaceae*, *Rosaceae*, *Rubiaceae*, *Ruppiaceae*, *Rutaceae*, *Salicaceae*, *Salviniaceae*, *Santalaceae*, *Sapindaceae*, *Sarraceniaceae*, *Saururaceae*, *Saxifragaceae*, *Scheuchzeriaceae*, *Schizaeaceae*, *Scrophulariaceae*, *Selaginellaceae*, *Simaroubaceae*, *Smilacaceae*, *Solanaceae*, *Staphyleaceae*, *Tamaricaceae*, *Taxaceae*, *Thelypteridaceae*, *Thymelaeaceae*, *Tofieldiaceae*, *Typhaceae*, *Ulmaceae*, *Urticaceae*, *Verbenaceae*, *Violaceae*, *Vitaceae*, *Woodsiaceae*, *Xanthorrhoeaceae*, *Xyridaceae*, *Zosteraceae*, *Zygophyllaceae*.

### Common names

Vascular plants, Lycopods, ferns, conifers, flowering plants. In the dataset, French and English vernacular names are provided for families, species, subspecies, and varieties.

## Spatial coverage

**Figure 2.**
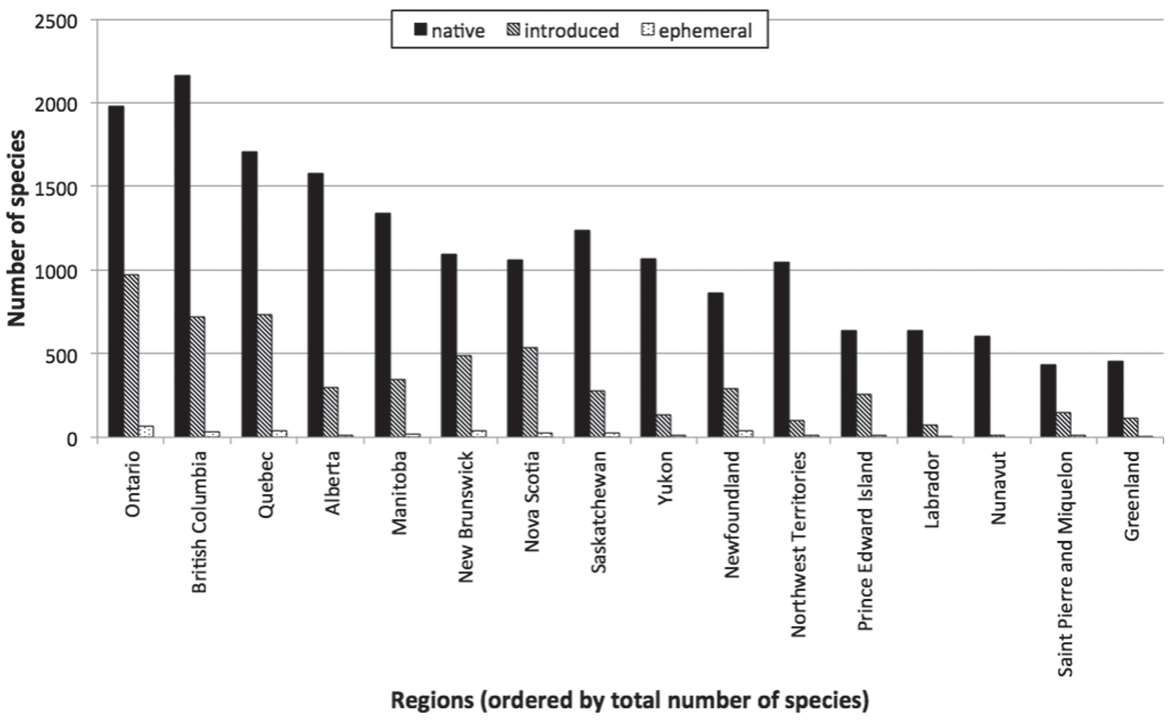
Regional distribution of accepted species from the Database of Vascular Plants of Canada (VASCAN). For each region, the number of native, introduced and ephemeral species is shown, i.e. species with a confirmed presence in the region. The regions are ordered by total number of species.

The checklist covers all vascular plants reported in Canada, Greenland (Denmark), and Saint Pierre and Miquelon (France) (Figure 2). The latter two regions are added because their floras are intimately related to that of Canada and it is useful for Canadians and others to know about them. Provincial distributions are provided to help Canadians visualize the relationship among the floras of their provinces and territories. VASCAN does not intend to replace regional or provincial lists but to act as a complement to them. The covered regions are, in alphabetical order: Alberta, British Columbia, Greenland, Labrador, Manitoba, New Brunswick, Newfoundland, Northwest Territories, Nova Scotia, Nunavut, Ontario, Prince Edward Island, Quebec, Saint Pierre and Miquelon, Saskatchewan, and Yukon.

The distribution status of the plant is indicated per region. These can be grouped as present (native, introduced or ephemeral), previously reported but currently considered absent (excluded, extirpated), doubtful or not reported (absent). The latter status is not recorded in the database (null value). Excluded taxa are those considered not currently occurring in a region, due either to non-recurring ephemeralness, misidentification, lack of supporting documentation, or when specimens are old and the taxon has not been observed again in more than 50 years. All distribution statuses are defined at http://data.canadensys.net/vascan/about/#distribution.

The VASCAN website (http://data.canadensys.net/vascan) provides a distribution map for each taxon. For higher taxa, these are calculated based on lower taxa, with the distribution statuses ordered as such: native, introduced, ephemeral, excluded, extirpated, doubtful, absent. E.g., if two species within the same genus are respectively native and doubtful in a certain region, the genus is considered native for that region.

The website also provides a checklist builder (http://data.canadensys.net/vascan/checklist), where users can generate their own list of taxa based on several criteria (taxonomy, region, distribution status, or a combination of these) and download this as a Darwin Core Archive or text file.

### Bounding box for covered area

41°40'N and 83°40'N latitude; 141°00'W and 11°19'W longitude

## Temporal coverage

17th to 21st century.

## Sampling methods

### Study extent description

See the section ‘Spatial coverage’ and ‘Project details - Study area description’.

### Sampling description

The data are sampled manually from literature by the editors, though recent additions are based on specimens maintained at institutional herbaria across Canada (see Thiers).

All floras covering Canada, Greenland, and Saint Pierre and Miquelon were considered for literature-based data entry, but only the most recent provincial and territorial floras (see the section ‘References - References used to build the dataset’) were systematically searched to establish the distribution status of each taxon in each region (see the section ‘Spatial coverage’). Scoggan’s Flora of Canada (1978-1979) was systematically searched, as were Kartesz (1999) and the Flora of North America (FNA Ed. Comm. 1993+). English and French vernacular names are based on usage in Canada and, for introduced taxa, on vernaculars from the countries of origin (when the taxon is from Europe). Alternate (synonym) vernaculars are provided when several names are in usage (notably regional names), but an accepted vernacular is recommended for general usage throughout the country. The method of selection of vernacular names follows Darbyshire et al. (2000). The source of the information is referenced for all scientific names, vernacular names and distributions in the dataset.

### Quality control description

New findings or corrections for plant distributions are communicated to the editors by contributors from each region (Appendix). Contributors are local botanists, often associated with Canadian herbaria or Conservation Data Centers. All new reports must be documented by specimens deposited at institutional herbaria.

Suggestions or corrections regarding names, taxonomy, or functionality of the VASCAN website are submitted by users and reviewers through a public Google Code issue tracker at http://code.google.com/p/canadensys/issues/list?can=2&q=label:vascan. Name suggestions are validated by the editors against names in Tropicos (http://www.tropicos.org), IPNI (http://www.ipni.org), GRIN (http://www.ars-grin.gov), or other plant name databases, before being manually corrected in the database.

## Dataset

The data are stored in a relational database (MySQL), which powers the search, checklist builder, taxon and name pages of the VASCAN website. Editors update a development copy of the database through a secure web application. This allows them to make revisions without affecting the users of the website. Once they agree that the data are consistent, in which they are aided by the application, they can push that version of the database to production.

**Figure 3.**
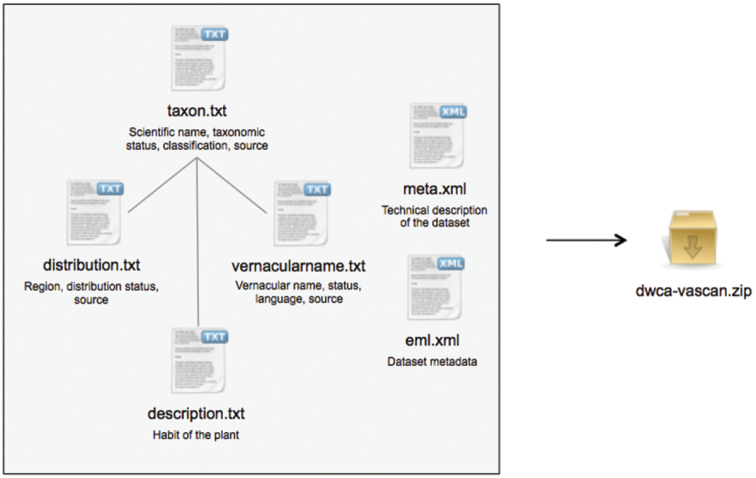
The VASCAN Darwin Core Archive, structured following the GBIF GNA Profile. It is a compressed folder containing 4 text files with tab-seperated values and 2 xml files. Taxon and scientific name information is provided in *taxon.txt*, with one record for each taxon and child-parent-relationships representing the classification. Records in the extension files *distribution.txt*, *vernacularname.txt* and *description.txt* have a many-to-one relation with the records in *taxon.txt* and provide additional information for each taxon. The archive structure and term definitions are described in *meta.xml*. The dataset metadata are provided in *eml.xml*.

At that moment, the application will also automatically generate a Darwin Core Archive of the data, using the GBIF GNA Profile (GBIF 2010) and following best practices for publishing checklists (GBIF 2011). This archive (Figure 3) includes all data, except for calculated distributions, hybrid parents, and user credentials. The archive is then manually uploaded to the Canadensys Repository (http://data.canadensys.net/ipt), a GBIF Integrated Publishing Toolkit, and republished, at which time it will be assigned a new version number (version 24 at the time of publication). The dataset is registered with the Global Biodiversity Information Facility (GBIF), which allows that organization to harvest, display and distribute the data at any time.

To the extent possible under law, the Université de Montréal Biodiversity Centre has waived all copyright and related or neighboring rights to this dataset, releasing it to the public domain under a CC0 waiver. Users of the data are encouraged to follow the Canadensys norms for data use and publication (http://www.canadensys.net/norms):

*Give credit where credit is due*: As is common practice in scientific research, cite the data you are using.

*Be responsible*: Use the data responsibly. The data are published to allow anyone to better study and understand the world around us, so please do not use the data in any way that is unlawful, harmful or misleading. Understand that the data are subject to change, errors and sampling bias. Protect the reputation of the data publisher and clearly indicate any changes you may have made to the data.

*Share knowledge*: Let us know if you have used the data. It helps us to showcase our efforts and it helps you reach a wider audience. Inform us if you have comments about the data, notice errors, or want more information.

*Respect the data license*: Understand and respect the data waiver under which the data are published. To help you make greater use of the data, we have dedicated the data to the public domain (CC0). Do not remove the public domain mark or provide misleading information about the copyright status.

**Object name:** Darwin Core Archive for the Database of Vascular Plants of Canada (VASCAN)

**Character encoding:** UTF-8

**Format name:** Darwin Core Archive format

**Format version:** 1.0

**Distribution:** http://dx.doi.org/10.5886/Y7SMZY5P

**Publication date of data:** 2013-07-22

**Language:** English

**Licenses of use:** http://creativecommons.org/publicdomain/zero/1.0/ & http://www.canadensys.net/norms

**Metadata language:** English

**Date of metadata creation:** 2013-07-22

**Hierarchy level:** Dataset

### Suggested citation for the latest version of the dataset

Brouillet L, Desmet P, Coursol F, Meades SJ, Favreau M, Anions M, Bélisle P, Gendreau C, Shorthouse D and contributors (2010+) Database of Vascular Plants of Canada (VASCAN). Online at http://data.canadensys.net/vascan, http://dx.doi.org/10.5886/1bft7W5f, and http://data.gbif.org/datasets/resource/13558, released on 2010-12-10. Version [xx]. GBIF key: 3f8a1297-3259-4700-91fc-acc4170b27ce. Data paper ID: doi: 10.3897/phytokeys.25.3100 [accessed on [date]]

## External datasets

**Object name:** GBIF data portal

**Character encoding:** UTF-8

**Format name:** various formats

**Distribution:** http://data.gbif.org/datasets/resource/13558

## Appendix

### Contributors

The following individuals made a significant contribution to VASCAN, in addition to the personnel listed in the section ‘Project details - Personnel’: G. Argus (Canadian Museum of Nature), S. Blaney (Atlantic Canada Conservation Data Center), B. Bennett (Environment Yukon), J. Cayouette (Eastern Cereal and Oilseed Research Centre), A. Cuerrier (Montreal Botanical Garden), R. Etcheberry (Saint Pierre and Miquelon), B. Ford (University of Manitoba), B. Fredskild (Denmark), K. Gandhi (Harvard University Herbaria), L. Gillespie (Canadian Museum of Nature), J. Gould (Alberta Tourism, Parks and Recreation), J. Greenall (Manitoba Conservation Data Centre), G. Halliday (Great Britain), C. Hanel (Newfoundland and Labrador Department of Environment and Conservation), V. Harms (University of Saskatchewan), S.G. Hay (Université de Montréal Biodiversity Centre), J. Labrecque (Centre de données sur le patrimoine naturel du Québec), F. Lomers (British Columbia), J. Maunder (The Rooms Provincial Museum, Newfoundland), M. Munro (Nova Scotia Museum), M. Oldham (Ontario Natural Heritage Information Centre), E. Punter (University of Manitoba), and J.C. Semple (University of Waterloo), as well as many people who made single contributions.
